# Supplementary material for: MacroGreen, a simple tool for detection of ADP-ribosylated proteins
Source: Commun Biol. 2021 Jul 28;4:919. doi: 10.1038/s42003-021-02439-w (PMC8319303; doi:10.1038/s42003-021-02439-w)
Supplement: Supplementary file 2 — Supplementary Information [file 42003_2021_2439_MOESM2_ESM.pdf]

Supplementary Note 1

MacroGreen, a simple tool for detection of ADP-ribosylated proteins

Antonio Ginés García-Saura[1], Laura K. Herzog[2,3], Nico P. Dantuma[2] & Herwig Schüler[1,4]

[1]Department of Biosciences and Nutrition, Karolinska Institutet, Huddinge, Sweden

[2]Department of Cell and Molecular Biology, Karolinska Institutet, Stockholm, Sweden

[3]Present address: Department of Chemistry, Umeå University, Umeå, Sweden

[4]Center for Molecular Protein Science, Department of Chemistry, Lund University, Lund, Sweden

Contents

Supplementary Figure 1: ADP-ribosyl hydrolase activity and ADP-ribose binding of Af1521-GFP mutant variants. 2

Supplementary Figure 2: Rationale for Af1521 mutagenesis. 3

Supplementary Figure 3: Affinity of MacroGreen for ADP-ribosylated target measured by SPR. 4

Supplementary Figure 4: MacroGreen detection of remaining ADP-ribosylation after ADP-ribosyl glycohydrolase and chemical treatment. 5

Supplementary Figure 5: MacroGreen staining of damage induced PARylation at double strand break sites. 6

Supplementary Figure 6: GFP staining of cells containing damage induced PARylation..... 7

Supplementary Figure 7: Detection of ADP-ribosylation in HEK293T cells after induced DNA damage. 8

Supplementary Figure 8: Quality control of MacroGreen by SDS-PAGE and Coomassie staining. 9

Supplementary Methods 10

Supplementary References 14

Supplementary note 11

Optimization of a microplate-based method for detection of ADP-ribosylation 11

Comparison of different protein binding plates 11

Figure 1: Optimization of a MacroGreen protein overlay assay protocol. 12

Figure 2: MacroGreen plate-based assay protocol. 13

Table 1: Statistical parameters of the MacroGreen plate-based assay* 14

References .... **Error! Bookmark not defined.**

Supplementary Note 1

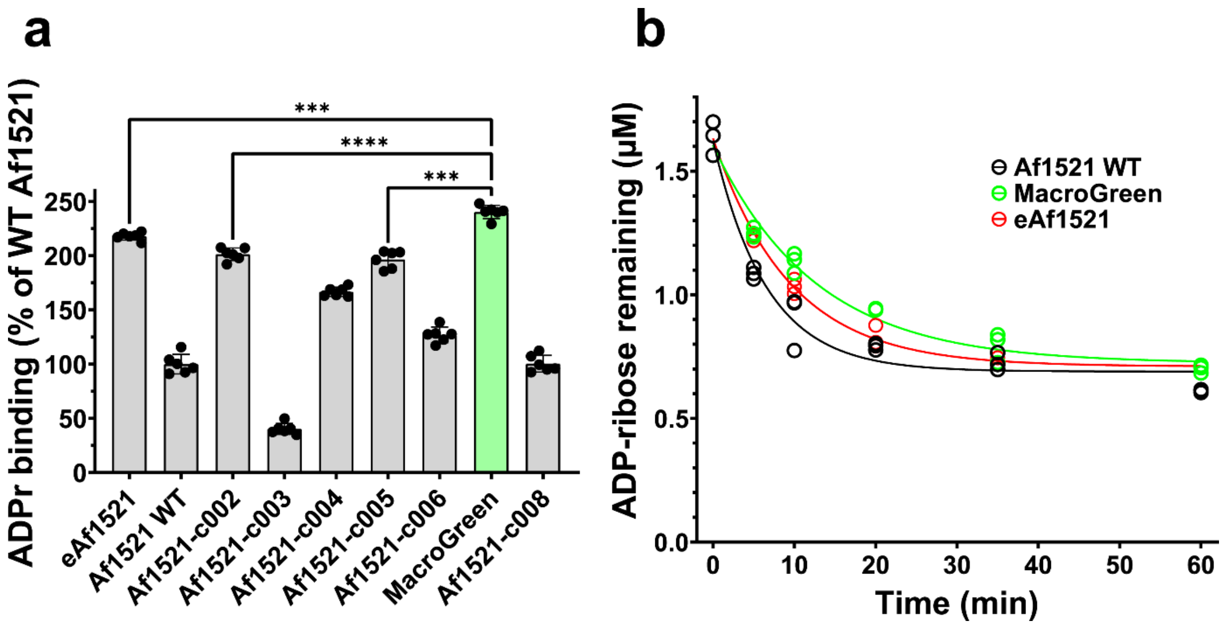

Supplementary Figure 1: ADP-ribosyl hydrolase activity and ADP-ribose binding of Af1521-GFP mutant variants.

a Binding of Af1521-GFP constructs to the auto-MARylated catalytic domain of PARP10. After incubation with NAD^+ for automodification, PARP10 (50 µl; 1 µM) was attached to the wells of 96-well Nunc MaxiSorp™ plates and then overlain with macrodomain-GFP fusion proteins (50 µl; 1 µM). Plotted are fluorescence signals measured, converted to percent of signal produced by the wild type GFP-fusion protein. *n* = 6; error bars indicate S.D See Materials and Methods for further details. **b** Comparison of the kinetics of ADP-ribosyl removal catalyzed by wild type Af1521-GFP, eAf1521-GFP, and MacroGreen (Af1521-c007). Remaining ADP-ribosylation levels were measured using 2 % biotinylated NAD^+ in the ADP-ribosylation reaction, and detection using HRP-conjugated streptavidin and a luminol reagent. *n* = 3; catalytic rates based on these data are given in **Table 1** of the main text.

Supplementary Note 1

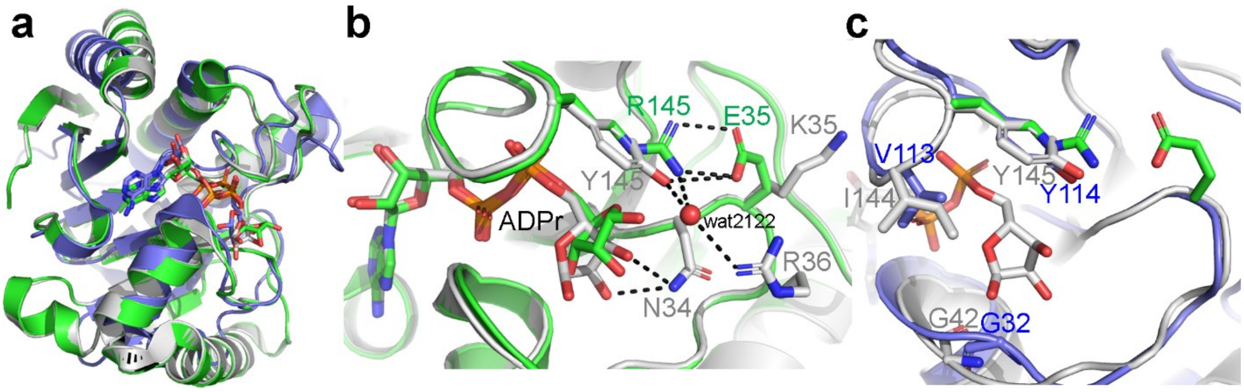

Supplementary Figure 2: Rationale for Af1521 mutagenesis.

a Overlay of the structural models of wild type Af1521 (grey carbons; PDB: 2bfq), eAf1521 (green; PDB: 6fx7), and the Chikungunya virus nsP3 macrodomain (blue; PDB: 6vuq) showing the structural similarity of the three proteins. All three crystal structures were determined with ADP-ribose bound. **b** Close-up of the active site and illustration of the residues that were probed by mutagenesis for their possible involvement in the catalytic mechanism. The water molecule was taken from the structure of the wild type protein (2bfq). Hydrogen bonds of relevance for the discussion in the main text are indicated as broken lines. Af1521 residues are labelled in grey and eAf1521 residues are labelled in green. **c** Close-up of the active site and illustration of residues probed based on published mutagenesis of nsP3. Chikungunya virus nsP3 residues are labelled in blue.

Supplementary Note 1

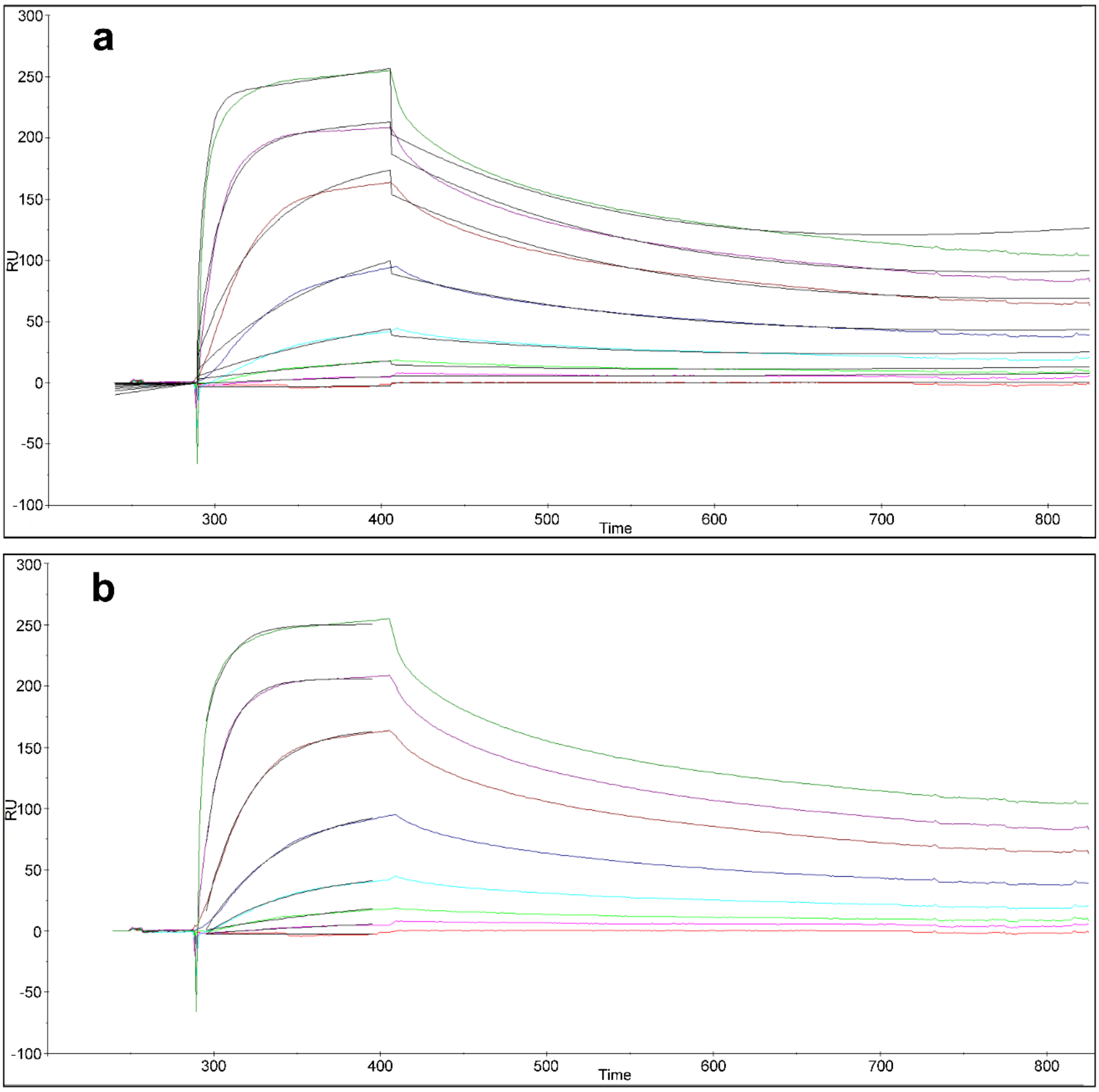

Supplementary Figure 3: Affinity of MacroGreen for ADP-ribosylated target measured by SPR.

Seven auto-MARylated PARP10 solutions (threefold dilutions at concentrations ranging from 1.11 µM to 1.52 nM) were used to probe binding to MacroGreen fixed to a CM5 chip. **a** Binding and unbinding traces and simultaneous k_a/k_d fitting with the 1:1 binding with drifting baseline model suggested K_D = 29.4 nM, χ^2 = 4.25. **b** Fitting of the curves to a 1:1 separate association (Langmuir) model suggested very similar affinity (K_D = 29.87 nM) but produced a better curve fit (χ^2 = 1.61).

Supplementary Note 1

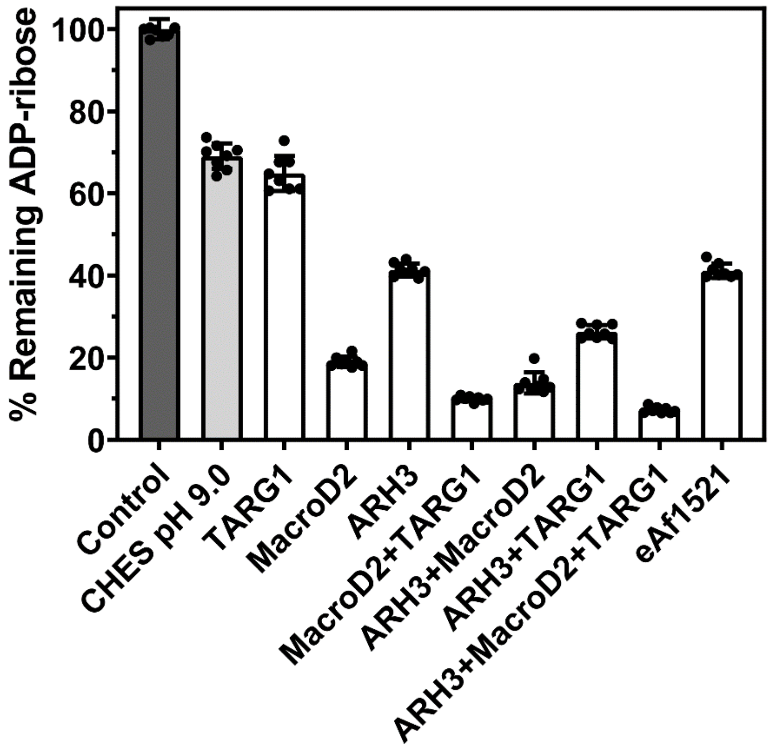

Supplementary Figure 4: MacroGreen detection of remaining ADP-ribosylation after ADP-ribosyl glycohydrolase and chemical treatment.

PARP10 protein was allowed to auto-MARylate before the reaction mixture was divided and aliquots were treated as indicated to remove the modification (further details are given in the Supplementary Methods section below). Then, the remaining levels of PARP10 auto-MARylation were quantified using MacroGreen fluorescence. PARP10 alone (control; dark grey) or after treatment with CHES buffer to remove carboxylate-linked MARylation (grey) or after treatment with side chain linkage specific glycohydrolases (white, as indicated). n = 8; error bars indicate S.D.

Supplementary Note 1

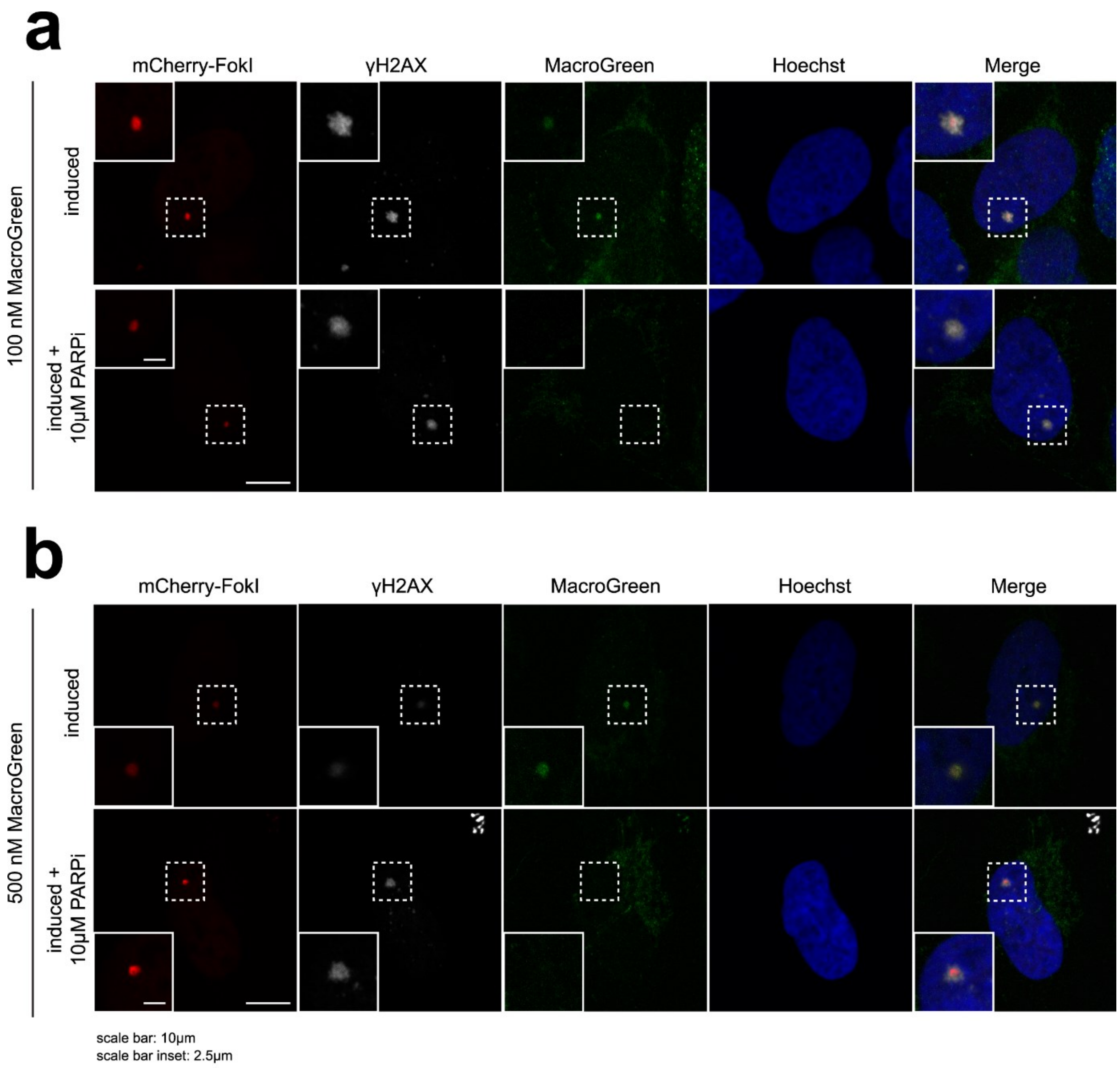

Supplementary Figure 5: MacroGreen staining of damage induced PARylation at double strand break sites.

U2OS DSB reporter cells were induced for 5 h using 1 µM Shield1 and 1 µM 4-OHT. PARP inhibitor was added where indicated at a final concentration of 10 µM (PARPi). Cells were stained for γH2AX. Cells were incubated with three concentrations of MacroGreen: **a** 100 nM; **b** 500 nM. Scale bar: 10 µm; scale bar inset: 2.5 µm.

Supplementary Note 1

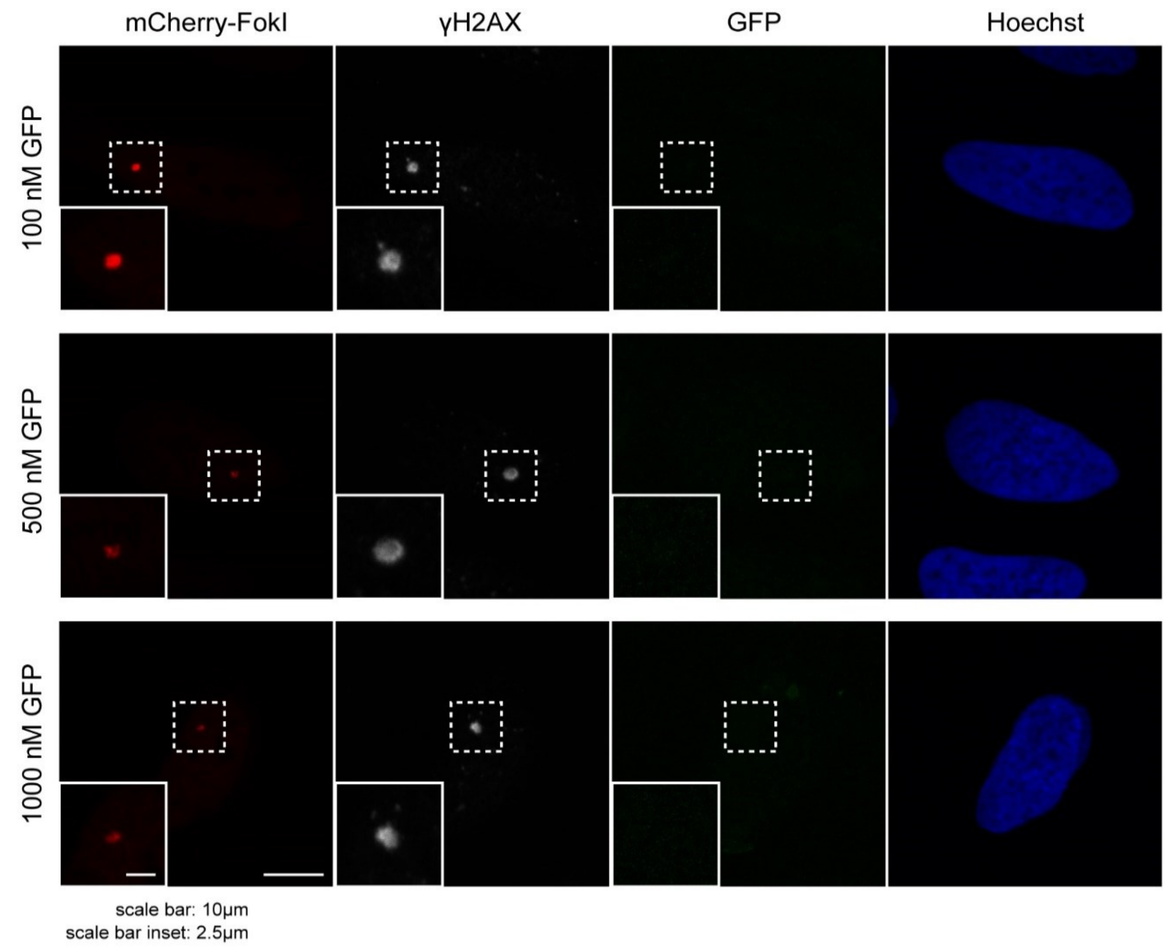

Supplementary Figure 6: GFP staining of cells containing damage induced PARylation.

Control experiments carried out as in **Supplementary Figure 5** above (no PARP inhibitor added), but staining cells with GFP instead of MacroGreen, at equivalent concentrations.

Supplementary Note 1

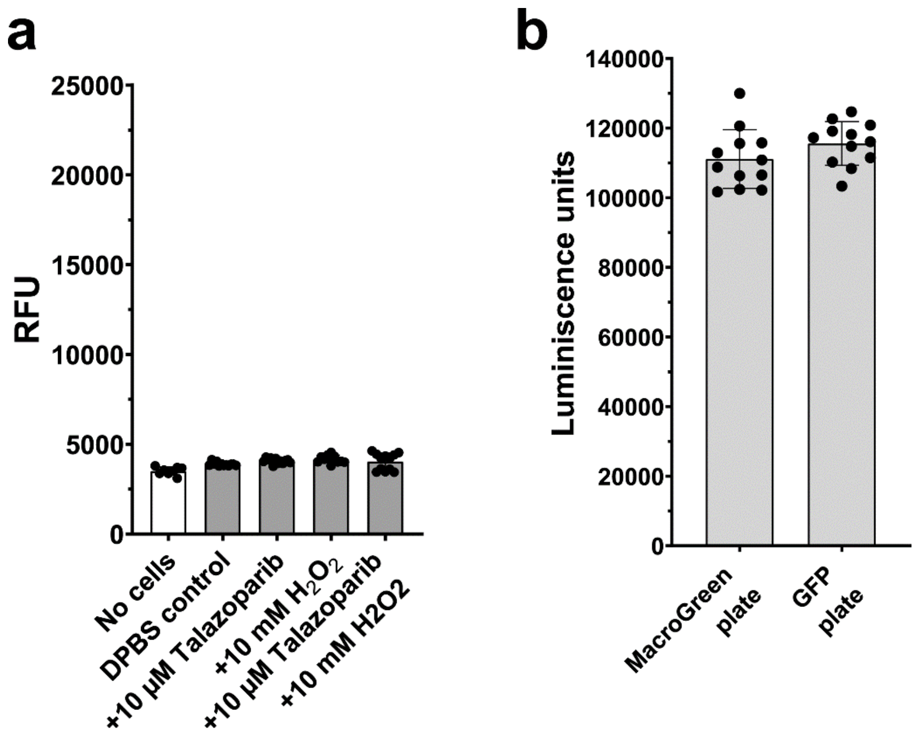

Supplementary Figure 7: Detection of ADP-ribosylation in HEK293T cells after induced DNA damage.

HEK293T cells were treated with 10 mM H_2O_2 to induce DNA damage in the presence or absence of PARP1 inhibitor Talazoparib. After cell fixation, the ADP-ribosylation levels were detected using MacroGreen (main text, Figure 4b). **a** shows the outcome of fixed cell staining with GFP, a negative control experiment. **b** An even number of cells on both plates was confirmed by processing two rows of cells per plate for ATP detection using CellTiter Glo reagent. Both panels: *n* = 12; error bars indicate S.D.

Supplementary Note 1

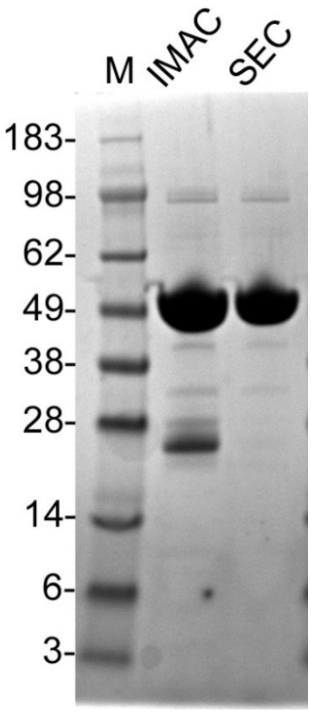

Supplementary Figure 8: Quality control of MacroGreen by SDS-PAGE and Coomassie staining.

After chromatography, pooled column fractions were adjusted to a protein concentration of 10 µM based on the theoretical extinction coefficient of the MacroGreen protein and absorbance measurements using a Nanodrop spectrophotometer (Thermo Fisher Scientific). Molecular weight marker (Invitrogen SeeBlue Plus2; 8 µl) and protein samples (20 µl including 5 µl 4x SDS-PAGE sample buffer) were loaded on 4-12% Bis-Tris gels (NuPAGE, Invitrogen) and separated by electrophoresis using MES-containing running buffer (Invitrogen). Gels were stained with CGP stain.[1] Shown are molecular weight marker (lane M), pooled IMAC peak fractions (IMAC), and pooled SEC peak fractions (SEC).

Supplementary Note 1

Supplementary Methods

ADP-ribosyl hydrolysis. To de-MARylate PARP10 catalytic domain protein, 50 picomoles (1 µM in 50 µL) of the respective enzyme (human recombinant ARH3, MacroD2, TARG1; or Af1521 wild type or mutants) were added to PARP10 treated as described above, and incubated for 60 min at RT under constant shaking.

Determination of statistical parameters for MacroGreen overlay assay. The Z-factor is a dimensionless parameter for quality assessment and development of new high-throughput screenings (HTS) assays. The Z-factor is defined in terms of four parameters: the means (µ) and standard deviations (σ) of both the positive/sample (p) and negative/background (n) controls (μ_p. σ_p. and μ_n. σ_n).[1] Given these values, the Z-factor is defined as:

$$Z - \text{factor} = 1 - \frac{(3\sigma p + 3\sigma n)}{(\mu p - \mu n)}$$

The percent coefficient of variation (%CV) is a measure of the dispersion of a probability distribution.[2] For the %CV calculation, σ and µ are the standard deviations and mean of MIN (background) or MAX (sample) control signals respectively:

$$\%CV = 100 * \frac{\sigma}{\mu}$$

Surface plasmon resonance measurements. SPR was performed at RT using a Biacore 2000 instrument (Biacore AB, Uppsala, Sweden). MacroGreen protein in SPR-PARP10 buffer (50 mM HEPES, 100 mM NaCl, 0.2 mM TCEP, 4 mM $MgCl_2$, 0.1 mM EDTA, pH 7.5) was immobilized on a CM5 chip by covalent cross-linking of amine groups to the carboxymethylated dextran matrix. The immobilization resulted in a surface density of 503.5 RU. In the binding experiment, three-fold dilution series of seven concentrations of auto-MARylated catalytic domain of PARP10 were injected for 120 s at a flow rate of 50 µL/min in SPR-PARP10 buffer. Auto-MARylated catalytic domain of PARP10 concentrations were in the range from 1111.11 to 1.52 nM. An uncoated flow cell 1 of the sensor chip was used as a reference. The data were evaluated using BIAevaluation software version 4.1.1. Sensorgrams were fitted using a 1:1 kinetic model with simultaneous k_a/k_d. Using only association sensorgrams, the data were also fitted to a Langmuir association model.

Supplementary Note 1

Optimization of a microplate-based method for detection of ADP-ribosylation

Since a plate-based assay to detect PARP enzyme activity and inhibition is likely the primary application for MacroGreen, we optimized a protocol for that application. First, we studied the time needed for maximum binding of auto-MARylated PARP10 to Nunc MaxiSorp^{TM} plates. After 5 minutes incubation at room temperature, 80% of the highest signal produced was achieved; and the highest signal was achieved after a 30 minutes incubation of the protein in the plates (**Figure 1a** below).

Next, we optimized the blocking step following the initial protein binding. The result showed that at a bovine serum albumin (BSA) concentration of 1 % w/v, the well surface was fully blocked after a 5-minutes incubation (**Figure 1b**). Longer blocking times caused a slight reduction in the fluorescence signal, possibly due to unbinding of ADP-ribosylated target protein.

Finally, we varied the time of incubation with the MacroGreen protein. The result showed that MacroGreen binding to plates coated with MARylated target is fast and efficient, producing superior signal-to-noise ratios after 5 minutes of incubation (**Figure 1c**). Longer incubation times caused a slight reduction in the fluorescence signal, and we speculate that this is a consequence of unbinding of ADP-ribosylated target protein, as in the previous step, in combination with remaining ADP-ribosyl glycohydrolase activity.

A schematic summary of a generic protocol for MacroGreen fluorescence detection of ADP-ribosylated proteins in protein binding multiplates is given in **Figure 2**. This protocol can be used as a starting point for optimization of an assay of ADP-ribosylation.

Comparison of different protein binding plates

We tested several commercial protein binding plates in the ADP-ribosylation assay described above. **Figure 1d** below shows a comparison of high protein binding plates (Nunc MaxiSorp^{TM}) with medium binding plates (Greiner Bio-One #655076), both in 96-well format. The results show that higher fluorescence signals were obtained in the high protein binding plates; and that both types of plate produced a wide linear range of signal increase. We also carried out assays in 384-well format with similar results and statistics (not shown). To conclude; comparison of different plate types and makes can aid in the optimization of a particular assay.

Supplementary Note 1

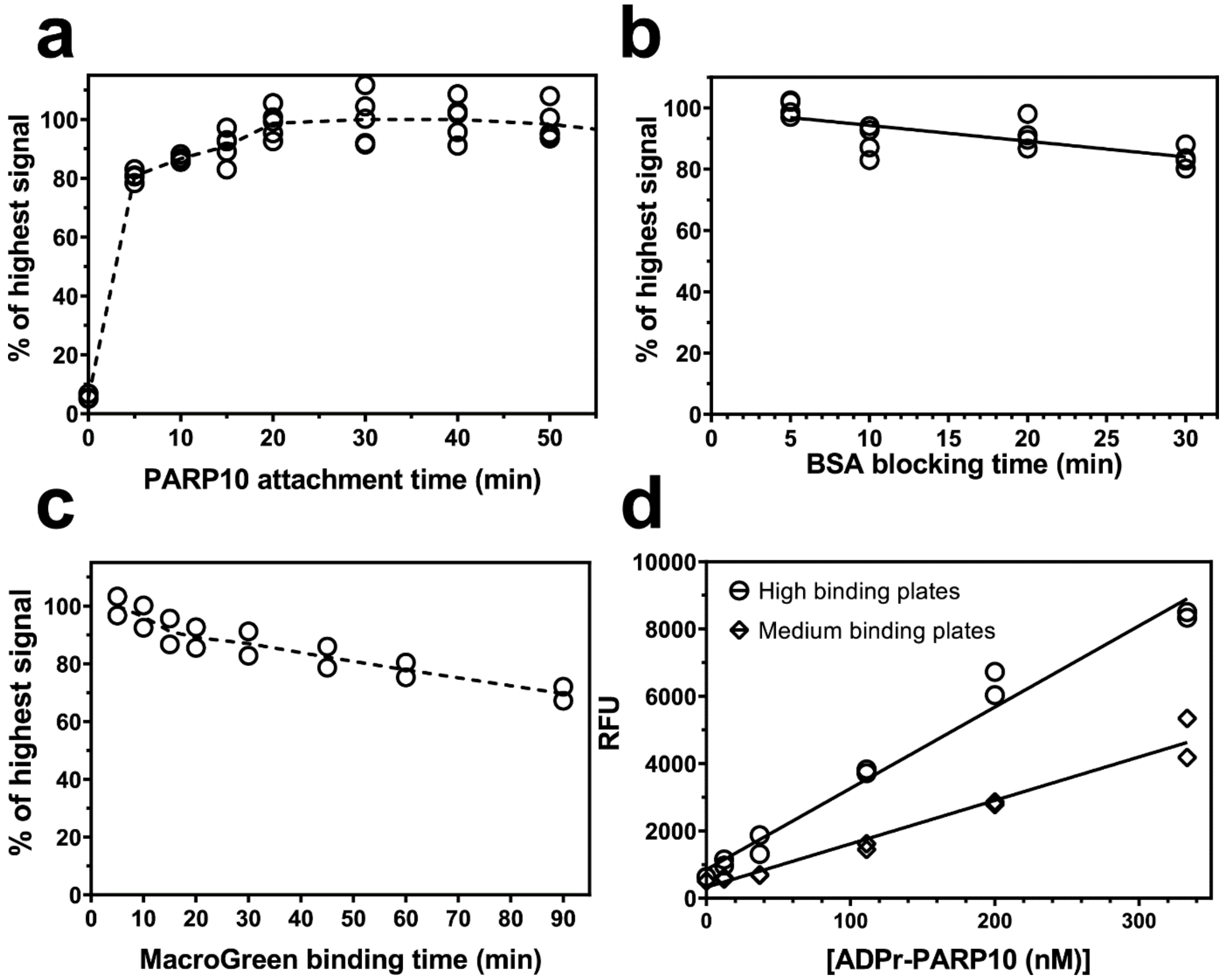

Figure 1: Optimization of a MacroGreen protein overlay assay protocol.

a MacroGreen fluorescence signal dependence on the time of attachment of auto-MARylated PARP10 to assay plates. *n* = 5. **b** Determination of the optimal BSA blocking step duration. *n* = 4. **c** Time of incubation with the MacroGreen protein was tested between 5 and 90 minutes. *n* = 2. **d** Comparison between two types of commercial protein binding plates. *n* = 2. All panels: error bars represent S.D. See text for details.

Supplementary Note 1

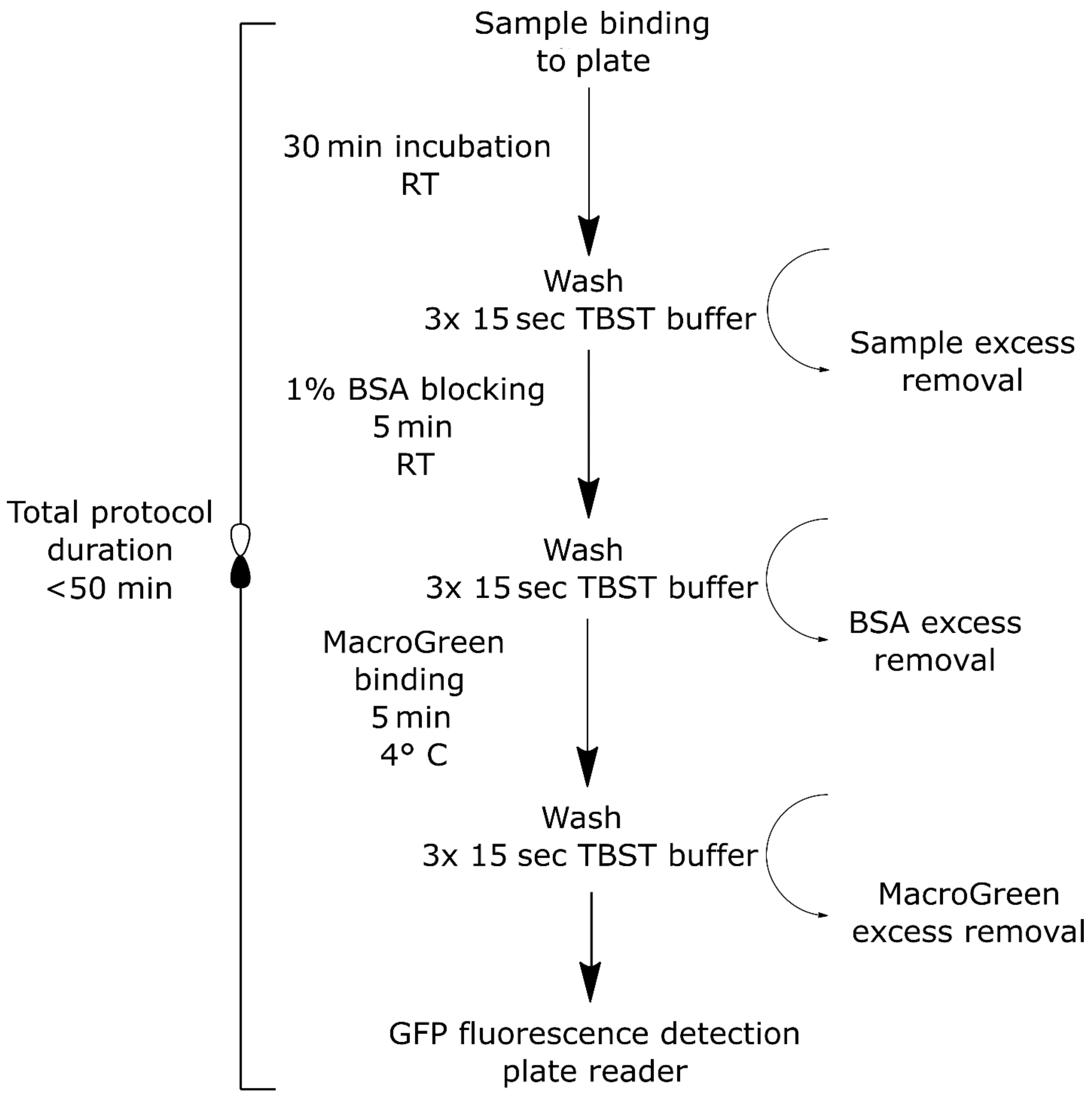

Figure 2: MacroGreen plate-based assay protocol.

Flowchart expanding the method used for protein overlay assays of MAR- and PARylated target proteins and detection using MacroGreen fluorescence.

Supplementary Note 1

Table 1: Statistical parameters of the MacroGreen plate-based assay*

ADP-ribosylated substrate	Working concentration [nM]	Z score	S/B ratio	MAX control %CV	MIN control %CV
	1000.0	0.90	124.56	3.40	4.45
	333.3	0.90	130.34	3.21	2.28
**PARylated PARP1**	111.1	0.93	131.61	2.20	4.45
	37.0	0.63	12.13	11.08	4.10
	12.3	0.48	1.79	3.98	6.50
	1000.0	0.95	17.85	1.43	3.77
	333.3	0.83	10.86	4.84	3.68
**MARylated PARP10**	111.1	0.85	5.66	3.69	2.46
	37.0	0.74	2.64	4.99	1.06
	12.3	0.32	1.65	6.41	4.25
	1000.0	0.78	5.57	5.11	4.64
	333.3	0.87	5.83	2.67	5.09
**MARylated actin**	111.1	0.80	6.14	4.71	5.92
	37.0	0.77	3.13	4.56	2.38
	12.3	0.54	1.91	4.33	5.65

*ADP-ribosylated target proteins (PARP1, PARP10 and actin at the indicated concentrations, 8 technical replicates each) were processed in 96-well MaxiSorp™ plates and probed with MacroGreen (1 µM). The table lists different high throughput screening assay quality descriptors based on the outcome of these assays, namely, the Z-factor,[1] signal-to-background (S/B) ratio, and the percent of coefficient variation (%CV).[2]

Supplementary References

1. Zhang, J.H., Chung, T.D. & Oldenburg, K.R. A Simple Statistical Parameter for Use in Evaluation and Validation of High Throughput Screening Assays. *Journal of biomolecular screening* **4**, 67-73 (1999).
2. Shun, T.Y., Lazo, J.S., Sharlow, E.R. & Johnston, P.A. Identifying actives from HTS data sets: practical approaches for the selection of an appropriate HTS data-processing method and quality control review. *Journal of biomolecular screening* **16**, 1-14 (2011).
3. Yasumitsu, H., Ozeki, Y., Kawsar, S.M., Toda, T. & Kanaly, R. CGP stain: An inexpensive, odorless, rapid, sensitive, and in principle in vitro methylation-free Coomassie Brilliant Blue stain. *Analytical biochemistry* **406**, 86-88 (2010).
