# Supplementary material for: MacroGreen, a simple tool for detection of ADP-ribosylated proteins
Source: Commun Biol. 2021 Jul 28;4:919. doi: 10.1038/s42003-021-02439-w (PMC8319303; doi:10.1038/s42003-021-02439-w)
Supplement: Supplementary file 3 — Description of Additional Supplementary Files [file 42003_2021_2439_MOESM3_ESM.pdf]

Description of Additional Supplementary Files

File name: Supplementary Data 1

Description: Experimental data underlying figure panels in the paper.
